# Comparative Study of the Structure, Composition, and Electrocatalytic Performance of Hydrogen Evolution in MoS*_x_*_~2+δ_/Mo and MoS*_x~_*_3+δ_ Films Obtained by Pulsed Laser Deposition

**DOI:** 10.3390/nano10020201

**Published:** 2020-01-24

**Authors:** V. Fominski, M. Demin, D. Fominski, R. Romanov, A. Goikhman, K. Maksimova

**Affiliations:** 1 Moscow Engineering Physics Institute, National Research Nuclear University MEPhI (Moscow Engineering Physics Institute), 115409 Moscow, Russia; dmitryfominski@gmail.com (D.F.); limpo2003@mail.ru (R.R.); 2 Russia Immanuel Kant Baltic Federal University, 236016 Kaliningrad, Russia; sterlad@mail.ru (M.D.); aygoikhman@gmail.com (A.G.); xmaksimova@gmail.com (K.M.)

**Keywords:** pulsed laser deposition, nanocatalysts, buffer gas, transition metal chalcogenides, hydrogen evolution reaction

## Abstract

Systematic and in-depth studies of the structure, composition, and efficiency of hydrogen evolution reactions (HERs) in MoS*_x_* films, obtained by means of on- and off-axis pulsed laser deposition (PLD) from a MoS_2_ target, have been performed. The use of on-axis PLD (a standard configuration of PLD) in a buffer of Ar gas, with an optimal pressure, has allowed for the formation of porous hybrid films that consist of Mo particles which support a thin MoS*_x_*_~2+δ_ (δ of ~0.7) film. The HER performance of MoS*_x_*_~2+δ_/Mo films increases with increased loading and reaches the highest value at a loading of ~240 μg/cm^2^. For off-axis PLD, the substrate was located along the axis of expansion of the laser plume and the film was formed via the deposition of the atomic component of the plume, which was scattered in Ar molecules. This made it possible to obtain homogeneous MoS*_x_*_~3+δ_ (δ~0.8–1.1) films. The HER performances of these films reached saturation at a loading value of ~163 μg/cm^2^. The MoS*_x_*_~3+δ_ films possessed higher catalytic activities in terms of the turnover frequency of their HERs. However, to achieve the current density of 10 mA/cm^2^, the lowest over voltages were −162 mV and −150 mV for the films obtained by off- and on-axis PLD, respectively. Measurements of electrochemical characteristics indicated that the differences in the achievable HER performances of these films could be caused by their unique morphological properties.

## 1. Introduction

Chalcogenides of transition metals, specifically, molybdenum sulfides (MoS*_x_*), form compounds with different local packings of metal (Mo) and chalcogen (S) atoms, which are organized into several crystalline and amorphous phases. If structures are crystalline, such as the layered 2H-MoS_2_ and 1T-MoS_2_ phases, which are nanometer sized, they can initiate a strong catalytic effect that activates a hydrogen evolution reaction (HER) during water electrolysis [1,2,3,4,5]. For this reason, thin-film molybdenum sulfide coatings are among the most used nonprecious nanoelectrocatalysts for hydrogen evolution. However, for widespread electrocatalytic applications, the HER activities and long-term stabilities of such nanomaterials are paramount. In the thermodynamically stable crystalline 2H-MoS_2_ phase, basal plane activation and edge site exposure/orientation on the cathode surface are the most frequently employed strategies [6,7]. The edge sites of the crystalline 2H-MoS_2_ phase exhibit the highest catalytic activity; however, in order to activate the basal planes, they should be modified by defect formation [8,9,10,11]. The catalytic activity of the metastable 1T-MoS_2_ phase does not depend so much on the orientation of the nanocrystals relative to the surface of the cathode; however, the synthesis of this phase is a difficult task [5].

Thin films of amorphous MoS*_x_* also have catalytic properties for HER activation. However, they require other approaches to enhance their activities [12,13,14,15]. Amorphous thin-film MoS*_x_* materials can greatly vary in their concentration of sulfur (1 ≤ *x* ≤ 10), which affects the local packing (chemical state) of atoms in the films. Currently, the factors that influence the catalytic activities of amorphous MoS*_x_* catalysts are being actively experimentally and theoretically studied [16,17,18,19,20]. An increased concentration of sulfur (*x* ≥ 2) and the formation of specific S ligands, and particularly the bridging and terminal S ligands in Mo_3_-S clusters, have been established to contribute to an increase in the catalytic activities of amorphous forms of MoS*_x_*. An adequate synergistic interaction of the amorphous MoS*_x_* film with the substrate/supporting material can also enhance the electrocatalytic HER activated by MoS*_x_* [21,22].

The most common methods for producing amorphous catalytic MoS*_x_* films are chemical and electrochemical synthesis/deposition [12,13,14,16,17,19]. The selection of precursors and the variation of the synthesis conditions makes it possible to obtain films with controllable morphologies, compositions, and chemical states. Advances in chemical synthesis have not yet resolved the urgency of the problem of obtaining analogical or more efficient amorphous electrocatalysts by the method of physical vapor deposition (PVD). The interest in this method is due to its environmental friendliness and the possibility of implementing other original conditions for the formation of new forms of amorphous MoS*_x_*.

Amorphous MoS*_x_* films began to be created intensively by PVD methods (i.e., ion sputter deposition and pulsed laser deposition (PLD)) more than 30 years ago, and their main use was related to the preparation of solid lubricating (low friction) coatings for the complicated working conditions of friction pairs [23,24,25,26]. Only very recently was the attention given to the potential of the use of PVD/PLD methods to obtain electrocatalytic films for HER activation [15,17,27,28,29]. In published articles, it has been reported that MoS*_x_* films obtained by the PLD method, under the standard configuration, possess sufficiently good electrocatalytic properties in terms of their reaction of hydrogen evolution. However, the task of these reports was not to determine the maximum attainable performance of MoS*_x_*-based electrocatalysts and to find the optimal conditions for the preparation of the most effective electrocatalyst by PLD. In particular, the problem of the possibility of variation of S content in a wide range, as well as the effect of the MoS*_x_*-based catalyst nanostructure and loading on the HER characteristics, have not been clarified.

However, changing the functionality of amorphous MoS*_x_* films requires significant modification in the conditions/regimes of their formation when using the PVD/PLD methods. For tribological application, MoS*_x_* films of a substoichiometric composition (*x* ≤ 2) were deemed most suitable. For application in the catalysis of HER, it is desirable to increase the concentration of sulfur significantly, and to implement such chemical states of amorphous MoS*_x_* films that will enhance the electrocatalytic performance of the created films. The use of reactive H_2_S gas with reactive PVD/PLD allows for variation of the S concentration in the formed MoS*_x_* films over a wide range [17,27]. However, this gas is dangerous, and the use of this gas causes technical and environmental problems. It is desirable to perform the PVD/PLD of MoS*_x_* films with designed characteristics using only MoS_2_ targets and nonreactive gas. These targets may be easily made from abundant earth material.

Also, for effective catalysis, it is important to obtain porous films that are not applicable in tribology. The peculiarity of pulsed laser ablation of MoS_2_ targets, which causes the formation of porous MoS*_x_* films, was revealed in the early studies of PLD concerning such films [30,31]. The originality of PLD from targets of transition metal dichalcogenides also lies in the formation of “core-in-shell” particles [32,33,34]. There is reasonable interest in investigating the influences of these factors on the electrocatalytic properties of the MoS*_x_* films obtained by PLD more deeply.

The use of a buffer gas during PLD alters the transport processes of the expansion of a laser plume from the MoS_2_ target and makes it possible to increase the S concentration in MoS*_x_* films above a stoichiometric value [29,35,36]. The actual composition of the deposited MoS*_x_* films depends on both the buffer gas pressure and the location of the substrate relative to the direction of the laser plume expansion. In cases where the substrate location is on the axis of the laser plume expansion and for locations perpendicular to it (i.e., using the on-axis PLD mode), all laser-ablated products, including the atomic flux and particles of submicron and nanometer dimensions, are deposited on the substrate. In cases where the substrate location is at a distance from the axis and parallel to it (i.e., using the off-axis PLD mode), the depositing flux consists of atoms that have been taken off from the target during ablation and scattered at large angles after collision with the buffer gas molecules.

Obviously, the on- and off-axis PLD methods have specific features in terms of their influences on the structures and chemical states of the deposited MoS*_x_* films. The objective of this work was to perform a rather in-depth study of the main characteristics of MoS*_x_* films prepared by on- and off-axis PLD, and to determine the highest performance of HER that can be achieved with MoS*_x_* films deposited on glassy carbon substrates by these methods. Within this aim, it was necessary to solve the problem of determining the optimal pressure of the buffer gas for the PLD of high performance MoS*_x_* films and to identify the criteria/features that allow for the appropriate selection of this parameter.

## 2. Materials and Methods

### 2.1. Experimental Methods for the on- and off-Axis PLD of MoS_x_ Films

**Figure 1:**
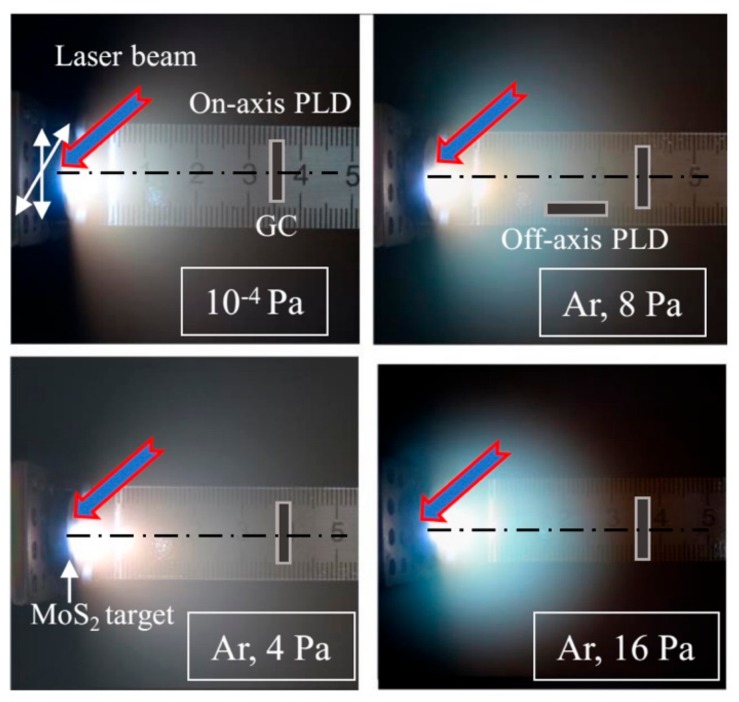
Time integrated optical images of the laser plume formed during the pulsed laser deposition of MoS*_x_* films from a MoS_2_ target under vacuum, considering various pressures of the Ar buffer gas. The arrangement of glassy carbon (GC) substrates, with respect to the axis of laser plume expansion, is shown for the modes of on- and off-axis pulsed laser deposition (PLD).

Figure 1 shows time-integrated pictures of laser plumes measured during the pulsed laser ablation of MoS_2_ targets under vacuum conditions and at different pressures of buffer gas (Ar). In the case of on-axis PLD in different conditions, the substrates were located at a normal angle to the axis of laser plume expansion and were 3.5 cm from the target. Off-axis PLD was performed at an Ar pressure of 8 Pa, and the substrates were located 2 cm from the target. The substrate surface was parallel to the axis of the laser plume expansion, and the shift of the substrate from this axis was ~0.3 cm. This setup precluded the influence of the shadow effect from the edge of the substrate on the distribution of the MoS*_x_* film over the surface of the substrate. The dimension of the substrate in the plane was approximately 0.7 × 0.7 cm^2^.

The MoS_2_ target was ablated by pulses from a Solar LQ529 laser (Solar, Minsk, Belarus). The laser beam fell at an angle of 45° to the surface of the target. The laser generation parameters were as follows: Radiation wavelength of 1064 nm, pulse duration of 15 ns, pulse energy of 14 mJ, laser fluence of 5 J/cm^2^, and pulse repetition rate of 50 Hz. The selected parameters provided a weak erosion of the target surface when exposed to a single laser pulse.

Preliminary studies from the authors have found that the use of laser radiation with a shorter wavelength of 266 nm from the ultraviolet region of the spectrum (the 4th harmonic of laser radiation) does not cause noticeable changes in the ablation future of the MoS_2_ target, as with the structure/composition of the deposited MoS*_x_* films.

A significant increase of energy in the laser pulse made it possible to increase the film growth rate; however, a strongly inhomogeneous distribution of the Mo and S elements over the substrate surface could be formed. In this case, in the center of the substrate, an area of metallization could arise [24]. An increase in the laser fluence, leading to strong ionization of vapors during the ablation of MoS_2_ target, results in a more isotropic expansion of the laser plasma. This could cause the formation of the most uniform distribution of the thickness/composition of the MoS*_x_* film over the surface of the substrate during on-axis PLD. However, the film growth rate decreases, due to the self-sputtering of the MoS*_x_* film by high-energy laser plasma ions. The preferential sputtering of S atoms by ions could result in the formation of a MoS*_x_* film with a very low S content (*x* ~1).

As illustrated in Figure 1, the target was moved in two directions, allowing for the maintenance of a relatively smooth surface of the target in the ablation zone under the repeatable laser ablation. The chamber for PLD was pumped with a turbomolecular pump to a pressure of ~10^−4^ Pa. The regime for the preparation of the MoS*_x_* films under this condition is indicated in the text as vacuum PLD. To conduct PLD in a buffer gas, after vacuum pumping, Ar gas was introduced into the chamber. The Ar pressure during the PLD ranged from 4 to 16 Pa. The deposition was performed in a small chamber with a volume of ~500 cm^3^, which made it possible to increase the partial pressure of the sulfur vapor during the prolonged ablation of the MoS_2_ target.

To analyse the character of the laser plume expansion under different vacuum conditions, plasma plume images were collected through the viewport (orthogonal to the axis of the plume motion) using a digital camera with an exposure time of 0.5 s. This time corresponded to an average value of more than 20 pulses. Different plumes were assumed to be equivalent, and therefore, the times, shapes, and sizes of the integrated plumes (i.e., the visible parts) were quite adequately recorded. In addition to the photoregistration of the pulsed laser plumes, measurements of the time-of-flight signal of a pulsed laser plasma were performed. The ion probe was placed 3.5 cm from the target. To reflect the electrons of the laser plasma, a negative bias of 40 V was applied to the probe.

### 2.2. Structural and Electrochemical Characterization of the Prepared MoSx Films

The MoS_x_ films with different thicknesses were deposited at room temperature on substrates made of polished glassy carbon (GC), polished silicon plates (including those with a layer of oxide (Si/SiO_2_)), and NaCl crystals. The time of PLD varied within the range of 1 min to 2 h. To measure the catalyst loading and to determine the S/Mo atom content ratio, Rutherford backscattering spectroscopy (RBS) was used (He ion energy of 1.3 MeV; scattering angle of 160°; ion beam diameter of 100 μm). Mathematical modelling of the RBS spectra was performed using the SIMNRA program.

The structures of the thin films deposited on the NaCl were studied using transmission electron microscopy and selected-area electron diffraction (TEM and SAED (selected-area electron diffraction), respectively, JEM-2100, JEOL, Tokyo, Japan). For this, the NaCl crystals with a deposited film were dipped in distilled water. After separation of the films from the substrate, the films were placed on fine-grained metal grids and transferred to an electron microscope. The structures, compositions and surface morphologies of the MoS*_x_* films prepared on the GC and Si substrates were studied using scanning electron microscopy with energy dispersive X-ray spectroscopy (SEM and EDS, Tescan LYRA 3), as well as micro-Raman spectroscopy (MRS), using a 632.8-nm (He-Ne) laser. The cross section of the laser beam was <1 μm. The chemical states of the MoS*_x_* films were studied using X-ray photoelectron spectroscopy (XPS, K-Alpha apparatus, Thermo Scientific, Madison, WI53711, USA) with Al Kα radiation (1486.6 eV). The Si/SiO_2_ substrates with deposited MoS_x_ films were split, and the fracture of the samples was studied with SEM. This procedure allowed us to examine the cross-sectional morphologies of the prepared films.

It should be noted that SEM and EDS studies of the MoS_2_ target have shown that the polished surface of the target was noticeably modified after irradiation with one laser pulse. However, the composition and morphology of the target surface formed after the first laser pulse were not modified substantially after prolonged laser ablation. This indicated that the main characteristics of the laser plume would not be changed during the deposition of the electrocatalytic MoS*_x_* films with growing loading.

The electrochemical studies of the MoS*_x_* films deposited on the GC substrates were performed in an H_2_-sparged 0.5 M H_2_SO_4_ aqueous solution using an Elins Instruments electrochemical analyzer (Model P-5X, Chernogolovka, Russia). A saturated silver chloride electrode (Ag/AgCl) was used as the reference electrode, and the GC with the pulsed laser-deposited MoS*_x_* film was the working electrode. For the used modes of on- and off-axis PLD, quite uniform distributions of the MoS*_x_* catalyst over the entire surface areas of the samples were achieved. All potentials reported in this work have been measured versus the reversible hydrogen electrode (RHE), and they were calculated according to the following formula: U(RHE) = U(Ag/AgCl) + (0.205 + 0.059 pH) (pH ~0.3). A bare GC plate was used as a counter electrode. For the electrochemical testing of a desired area on the GC substrate, the sample was placed in a special holder that was made of Teflon.

The main electrochemical characteristics of the MoS*_x_* films that were accepted for use in determining the performances of the HER catalysts included the measurements/evaluations of the cathodic polarization curves, turnover frequencies (TOFs) of the HER, double-layer capacitances (C_dl_), electrochemical impedances, and long-term stabilities. Explanations of the techniques of the electrochemical measurements that were used to study the MoS*_x_*-based catalysts can be found in some published works, e.g., References [14,37,38].

The cathodic polarization curves were measured using linear voltammetry (LV), with a sweep of the applied potential from 0 to −350 mV and a scan rate of 2 mV/s. Before the LV measurements, all samples were subjected to cathodic pre-treatment at −350 mV. The optimal catalyst loading was determined in accordance with the minimum overvoltage (*U*_10_) that was needed to achieve a current density of *j*_10_ = 10 mA/cm^2^.

The procedure for calculating the TOF of HER has previously been proposed [16,38]. Cyclic voltammograms (CV) were measured after applying the cathodic polarization of the sample in the same electrolyte in the potential range from 0 to 1200 mV. Integration of the current peaks which resulted from the oxidation of the active sites allowed for the measurement of the number of active sites on the catalyst surface. The relation of the current density (*j*), measured by LV, to the number of catalytically active sites allowed for the evaluation of the TOF of the catalyst using the expression TOF = *j*/2*Q*, where *Q* is the total charge. This charge was determined by integrating the current (*i*), as measured by CV; specifically, $Q\;=\;\frac{1}{U_{\mathrm{sr}}}\int_{E_{1}}^{E_{2}}idE,$ where *E* is the potential and *U*_sr_ is the potential scan rate during the CV measurements (50 mV/s).

For the estimation of *C*_dl_, the current densities versus the potential data were acquired by CV, while sweeping the applied potential at various scan rates (40–200 mV/s) in a potential range (30–140 mV), within which no Faradaic electron-transfer processes were observed. The double-layer capacitances of the MoS*_x_* films were calculated as the slopes of plots of the scan rates versus the current densities at 100 mV. Electrochemical impedance spectroscopy (EIS) was performed at an overpotential of −180 mV in the frequency range of 10^5^ to 10^−1^ Hz, with a perturbation voltage amplitude of 20 mV. All LV curves measured in this work were *iR*_s_-corrected. The equivalent series resistance (*R*_s_) was extracted from the EIS data. The stabilities of the prepared films were tested by continuously cycling the voltage between 100 and −350 mV at a scan rate of 50 mV/s.

## 3. Results

### 3.1. Selection of the Conditions (Buffer Gas Pressure) for the PLD of MoS_x_ Films

The dynamics of a laser plume in a vacuum are characterized by a practically free and collision-less propagation regime. The spatial distribution is strongly forward directed, and the observed light emission is weak. If a background gas is present in the deposition chamber, the light emission of the plume increases, due to particle collisions. These collisions produce radiative de-excitation of the ablated species, both in the body of the plume and particularly in the expansion front. The plume edge can be better defined, due to the presence of a shock wave front. The plume is slowed down and spatially confined. The time-integrated visible plume length might relate to the maximum distance reached by the shock wave front (i.e., the stopping distance) from the ablated target. Obviously, the composition, structure, and functional properties of the deposited films can significantly depend on both the buffer gas pressure and the location of the substrate, relative to the stopping distance of the laser plume [39,40].

**Figure 2:**
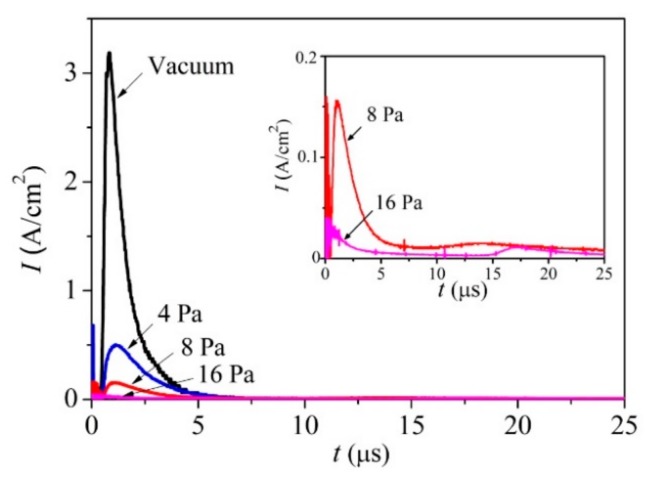
Pulsed ion signals that were detected by an ion probe during the pulsed laser ablation of the MoS_2_ target, both in a vacuum and at different pressures of Ar buffer gas.

Figure 1 shows that, in a vacuum and at an Ar gas pressure of 4 Pa, the laser plume expanded over the large volume of the chamber and enveloped the substrate for the deposition of the film in the on-axis PLD mode. At a pressure of 8 Pa, a noticeable limitation of the plume volume was observed, but the front of its expansion reached the substrate located for the on-axis PLD. At a pressure of 16 Pa, the stopping distance of the laser plume was less than the distance from the target to this substrate. Measurements of the ion signals of the pulsed laser plasma revealed that, for the ion flux bombarding the substrate, an increase in Ar pressure caused both a decrease in the intensity and a change in the ion energy (Figure 2). At a pressure of 8 Pa, along with a peak from the high-speed ions (time of ion flight up to 5 μs), a peak with a time of ion flight of more than 10 μs appeared. Moreover, at the pressure of 16 Pa, the time of flight for the main ion flux reaching the substrate increased to 15 μs.

Wood et al. [41] proposed that during ablation in a buffer gas, the laser plume is broken into orders corresponding to the numbers of collisions made with the background. The first order reaches the detector without any scattering, the second order undergoes one scattering event, and so forth. Scattered atoms move mainly by the mechanism of collective motion with the buffer gas molecules captured by the laser plume, i.e., those in the shock wave. Analyses of the results of the laser plume optical recording and the ion pulse measurement suggest that, at 8 Pa, a shock wave was indeed formed, but some of the atoms in the laser plume reached the substrate without scattering. This finding indicated that the formation of the MoS*_x_* films proceeded, due to the deposition of a scattered atomic flux. However, the flux-containing atoms from the second scattering order could retain the energetic motion toward the substrate, and therefore, could ensure good adhesion of the films to the substrate. For the higher pressure of 16 Pa, the formation of the MoS*_x_* film was proceeded by the deposition of atoms that had experienced multiple scattering events on the gas molecules and had lost the energy of directed motion to the substrate.

It is shown below that a pressure of 8 Pa was optimal for obtaining a highly efficient HER catalyst using the on-axis PLD mode. We considered this fact and also analyzed the optical images of the laser plume (Figure 1). The images indicated that, at the pressure of 8 Pa, there was no noticeable gradient of brightness along the axis of the expansion of the plume in the range of 2 to 3 cm from the target. This made it possible to obtain rather uniform MoS*_x_* films by off-axis PLD on substrates of up to 1 cm in size.

### 3.2. Deposition of the MoS_x~2+δ_/Mo Films by on-Axis PLD

**Figure 3:**
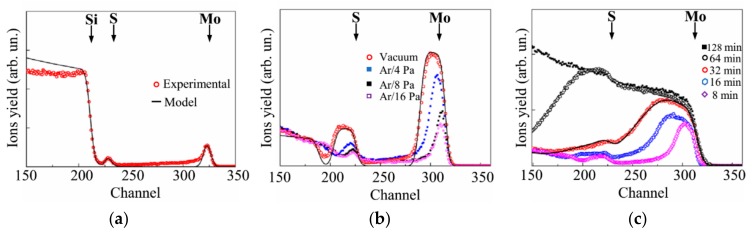
Rutherford backscattering spectroscopy (RBS) spectra for MoS*_x_*_~2+δ_/Mo films obtained by on-axis pulsed laser deposition (PLD) on the Si and Si/SiO_2_ substrates. (**a**) A thin film deposited for 1 min on the Si substrate. (**b**) Films of middle thickness deposited on the Si/SiO_2_ substrates for 4 min in a vacuum and at different Ar pressures. (**c**) Thicker films deposited on the Si/SiO_2_ substrate in Ar at a pressure of 8 Pa for various deposition times. The solid lines indicate the model/fitted RBS spectra for the corresponding experimental spectra. The arrows indicate the channels of accumulation of He ions which were back scattered by Mo, S, and Si atoms located on the surface of the samples.

Figure 3a shows the experimental and model RBS spectra for a thin MoS*_x_* film obtained on a Si substrate over 1 min of on-axis PLD at an Ar pressure of 8 Pa. According to the SIMNRA simulation, the composition of this film was MoS_2.7±0.2_. The loadings of the model thin-film catalyst were ~6.6 × 10^15^ Mo atom/cm^2^ (1.1 μg/cm^2^) and ~17.8 × 10^15^ S atom/cm^2^ (0.95 μg/cm^2^). At a density of 5.06 g/cm^3^, the film thickness was ~4 nm.

Notably, the actual load was greater, due to the Mo nanoparticles, which caused a long “tail” in the RBS spectrum (channels 240–315). Calculation of the “Mo peak/Mo tail” intensity ratio revealed that the Mo content in the thin MoS*_x_* film was approximately equal to that of the Mo nanoparticles. The total MoS_2.7_/Mo catalyst load was estimated to be ~3.4 μg/cm^2^, corresponding to a deposition rate of ~3.4 μg/cm^2^/min. For the used energy of the analyzed ion beam, the imposition of the RBS peak from the Mo atoms on the RBS peak from the S atoms occurred when the He ions were scattered by Mo particles larger than 230 nm. The use of the buffer gas reduced the deposition rate of the atomic component of the laser plume; however, this allowed for an increase in the S concentration in that component of the hybrid MoS*_x_*_~2+δ*/*_Mo films, which was formed, due to the deposition of the atomic flux.

Figure 3b shows the RBS spectra for the MoS*_x_*_~2+δ*/*_Mo films deposited on the Si/SiO_2_ substrate under different conditions for 4 min. These spectra indicated that the relative contribution of the Mo peak can be attributed to “Mo atoms in the MoS*_x_*_~2+δ_ film”, which reduced with increasing Ar pressure, where the yield of He ions in the range of channels 275 to 240 did not depend on the Ar pressure, which implies that the deposition flux of Mo particles persisted unchanged under various conditions of the on-axis PLD.

Due to the greater deposition rate of atomic flux during vacuum on-axis PLD, the RBS spectrum for a corresponding MoS*_x_*_~2+δ_ film could be adequately processed by SIMNRA if the model object consisted of a homogeneous MoS*_x_*_=2.1-0.2_ film on a SiO_2_ substrate. The catalyst loading included ~1.7 × 10^17^ Mo atom/cm^2^ (27.3 µg/cm^2^) and ~3.6 × 10^17^ S atom/cm^2^ (19.4 µg/cm^2^). The deposition rate for the vacuum on-axis PLD of the MoS*_x_*/Mo catalyst was 11.7 μg/cm^2^/min.

Figure 3c shows the dependence of the loading of the MoS*_x_*_~2+δ_/Mo catalyst on the time of its on-axis PLD in Ar at a pressure of 8 Pa. A monotonic growth of the catalyst loading was observed with increases in deposition time. The shapes of the RBS spectra are strongly distorted, due to the accumulation of Mo particles. An example of the mathematical fitting of the RBS spectra is shown in Figure 3c for a sample obtained after 32 min of on-axis PLD deposition. A fairly good match was obtained for a model that contained a thin surface layer with the composition MoS*_x_*_~1.8_ and a thicker underlayer with the composition MoS*_x_*_~1.4_. A satisfactory fit was achieved, due to the assumption of large surface roughness for the model film. The model film contained ~4.8 × 10^17^ Mo atom/cm^2^ (76 μg/cm^2^) and ~7.5 × 10^17^ S atom/cm^2^ (44 μg/cm^2^). The calculated deposition rate of the MoS*_x_*_~2+δ*/*_Mo film was 3.75 µg/cm^2^/min, which correlated well with the data regarding the deposition rate at the initial stage (i.e., for 1 min).

The spectra of the MoS*_x_*_~2+δ_/Mo films obtained with longer deposition times were difficult to process with SIMNRA (Figure 3c). Below, we show that the optimum catalyst loading was formed with an on-axis PLD time of 64 min. We assumed that, for this time of on-axis PLD, the catalyst loading increased to 240 µg/cm^2^ and included ~9.6 × 10^17^ Mo atom/cm^2^ (152 µg/cm^2^) and ~1.5 × 10^18^ S atom/cm^2^ (88 µg/cm^2^). After an increase in deposition time of up to 128 min, the RBS spectrum of the MoS*_x_*_~2+δ_/Mo film corresponded to a thick layer of a homogeneous mixture of Mo and S, and the atomic ratio of these components was S/Mo ≤ 1.

**Figure 4:**
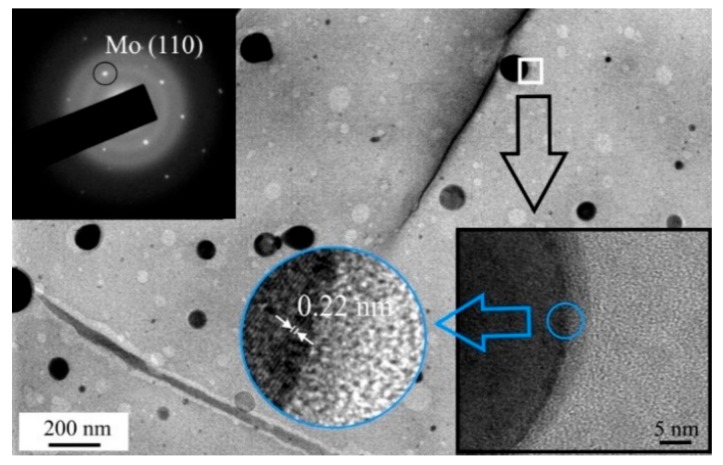
TEM image and selected-area electron diffraction (SAED) pattern of the thin MoS*_x~_*_2+δ_/Mo thin film obtained by on-axis PLD for 1 min in Ar at a pressure of 8 Pa. The bottom inserts show high resolution TEM images of the film.

The formation of Mo nanoparticles during the pulsed laser ablation of MoS_2_ targets and their transfer to the surfaces of the MoS*_x_*_~2+δ_/Mo films during the on-axis PLD was confirmed by the TEM, and MD results from a thin film deposited on NaCl (Figure 4). At low magnification, round-shaped dark particles were detected. The sizes of these particles varied in the range of 10–200 nm. The MD pattern of these particles corresponded to the cubic lattice of Mo. In high-resolution TEM imaging of a separate Mo nanoparticle, atomic planes with an interplanar distance of 0.22 nm, characteristic of Mo (110), were observed. The Mo particles were surrounded by an amorphous ~5 nm-thick shell. The MoS*_x_*_~2+δ_ matrix of the MoS*_x_*_~2+δ_/Mo film was amorphous. The TEM image of this film contained rounded nanosized areas with lighter contrast. These areas probably corresponded to the sites at which the Mo nanoparticles were localized. However, when manipulating the film for the TEM studies, these particles were removed, due to weak adhesion to the thin MoS*_x_*_~2+δ_ matrix.

**Figure 5:**
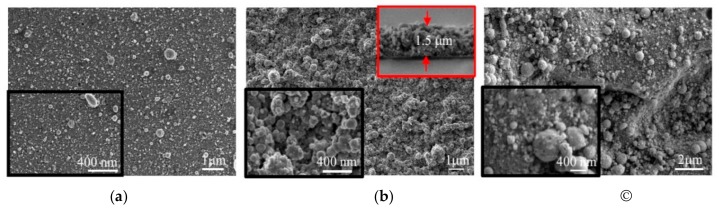
SEM images of MoS*_x_*_~2+δ_/Mo films obtained on the (**a**) Si, (**b**) SiO_2,_ and (**c**) glassy carbon (GC) substrates by on-axis PLD in Ar at a pressure of 8 Pa. The deposition times were (**a**) 4 min, (**b**) 64 min, and (**c**) 64 min. (**b**) The top inset shows a cross-sectional image of the film.

Figure 5a shows SEM images of a relatively thin MoS*_x_*_~2+δ_/Mo film deposited by on-axis PLD onto the smooth surface of the Si/SiO_2_ substrate. Here, the deposition time was 4 min, and at this stage of film growth, the film had a relatively dense structure. This structure was formed from sufficiently small Mo nanoparticles and a scattered flux of Mo and S atoms. The sizes of most of the Mo nanoparticles did not exceed 200 nm, and the scattered flux of Mo and S atoms provided a conformal deposition of a MoS*_x_*_~2+δ_ shell on these particles and enveloped them with approximately the same efficiency over the entire rounded surface.

Figure 5a shows that larger particles occasionally appeared in the surface of the MoS*_x_*_~2+δ_/Mo film. The sizes of these particles reached several fractions of a micrometer. The deposition of Mo particles of a submicron size had a significant effect on the formation of the morphologies of the thicker films. Figure 5b shows SEM images of the MoS*_x_*_~2+δ_/Mo film deposited on the Si/SiO_2_ substrate for 64 min. The large conglomerates are formed from submicro- and nano-sized particles, and the structure of the film became porous here. The film thickness, as estimated based on the SEM images of the cleaved Si/SiO_2_ substrate covered with this film, was ~1.5 μm.

An SEM image of the MoS*_x_*_~2+δ_/Mo catalyst obtained under the same conditions by on-axis PLD on the glassy carbon is shown in Figure 5c. The image indicates that the catalyst consisted of loose packaging of nano- and sub-micro-particles. The catalytic film covered the surface of the glassy carbon with a continuous layer. The GC substrate had noticeable roughness, and the lateral sizes of the surface cavities on this substrate were approximately several μm, and their depths varied in the range of ~0.1–1 μm. Due to the roughness of the glassy carbon substrate, the local loading of the MoS*_x_*_~2+δ_/Mo catalyst was somewhat less than 240 µg/cm^2^. EDS analysis of the sample, shown in Figure 5c, indicated that the concentrations of the Mo, S, O, and C atoms were 10.6, 10.2, 5.2, and 74%, respectively. A surface area of 10 × 10 μm^2^ was analyzed by EDS. The content of O atoms was practically unchanged when the EDS analysis was performed for a pure polished GC substrate. This result indicated that the O atom concentration in the films did not exceed several percent. The result of the EDS measurement of the ratio S/Mo in the thick MoS*_x_*_~2+δ_/Mo catalytic film coincided well with the result of its measurement by the RBS.

**Figure 6:**
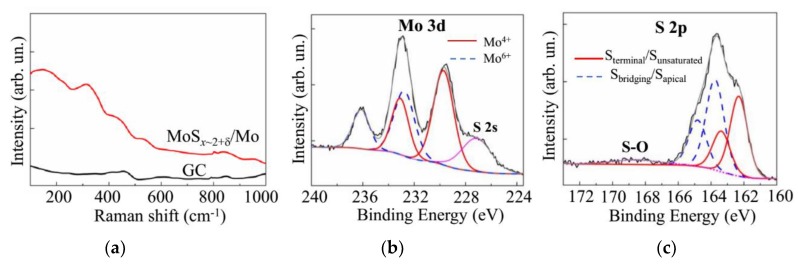
(**a**) Raman spectra for GC substrate, with and without the MoS*_x_*_~2+δ_/Mo thin film catalyst obtained by on-axis PLD. (**b**,**c**) XPS spectra of Mo 3d and S 2p, measured on the surface of MoS*_x_*_~2+δ_/Mo catalyst after prolonged exposure in the air.

Figure 6a shows the micro-Raman spectrum for a MoS*_x_*_~2+δ_/Mo catalyst obtained on glassy carbon by on-axis PLD for 4 min in Ar at 8 Pa. For this spectrum, there were no peaks characteristic of the MoS_2_ compound. Measurements of the Raman spectrum for the MoS_2_ target showed that the characteristic and most intense E_2g_^1^ and A_1g_ peaks were located at frequencies of 383 cm^−1^ and 408 cm^−1^, respectively. This spectrum confirms the amorphous structure of the catalytic layer, which is characterized by the presence of several broad vibrational bands near the frequencies of 200, 330, 450, and 540 cm^−1^. The same Raman data, with four very weak and broad bands, were obtained from MoS*_x_* films grown by traditional PLD elsewhere [42]. McDevitt et al. [42] proposed that this Raman spectrum indicates that the laser-deposited films represent a mixture of small domains of MoS_2_ and amorphous sulfur. It is more reasonable to use a more recent model of a cluster-based polymeric structure that consists of Mo_3_-S clusters with some different configurations of S ligands [4,16,18,27]. In the frame of this model, the characteristic Raman spectrum of amorphous MoS*_x_* contains the following vibration modes: ν(Mo-Mo) at ~200 cm^−1^, ν(Mo-S)_coupled_ at ~320 cm^−1^, ν(Mo-S_apical_) at ~450 cm^−1^, ν(S-S)_terminal_ at ~520 cm^−1^, and ν(S-S)_bridging_ at 540 cm^−1^. The Raman spectra of the MoS*_x_*_~2+δ_/Mo catalyst contained bands that could be attributed to all these modes. However, the vibration peaks of the bridging S_2_^2−^ and terminal S_2_^2^ moieties overlapped, which caused the appearance of a broadened band in the 500–560 cm^−1^ range. This finding indicated a weak order of atom packing in the local regions, comparable to the sizes of the Mo_3_-S clusters.

The spectrum of the film obtained by on-axis PLD contained no peaks that are characteristic of nanocrystalline molybdenum oxides. In the case of the formation of MoO_3_ nanocrystals, the distinctive peaks at ~820 and ~990 cm^−1^ were observed [18]. For the MoO_2_ nanocrystals, vibrations at ~205, 229, 345, 365, 498, 572, and ~745 cm^−1^ are characteristic [43]. However, the appearance in the spectrum in Figure 6a, with wide vibration bands at ~820 and ~950 cm^−1^, indicates the formation of disordered MoO_3-*y*_ clusters in the MoS*_x_*_~2+δ_/Mo film.

This conclusion was confirmed by the results of the XPS studies of the MoS*_x_*_~2+δ_/Mo film, which are shown in Figure 6b, c. The measurements of the XPS spectra were performed after the prolonged exposure of the film in the air (for approximately six months). The film was prepared by the on-axis PLD method in Ar at 8 Pa for 64 min. In the spectrum of Mo 3d, in addition to the doublet Mo 3d_5/2_‒Mo 3d_3/2_, which corresponds to the chemical bonding of Mo with S (Mo^4+^, the binding energy E_B_ of Mo 3d_5/2_ is 229.7 eV), there was a doublet found that was attributable to Mo oxide (Mo^6+^, Mo 3d_5/2_ E_B_~232.8).

The XPS studies of the MoS_2_ target indicated that, in the case of effective Mo-S bond formation, the surface of the compound had a higher resistance to oxidation in the air (results not shown). The formation of the molybdenum oxide nanophase could have resulted from the ineffective interaction of Mo and S atoms during the film deposition. Unsaturated Mo bonds in the local structure of the MoS*_x_*_~2+δ_ film interacted with O atoms when the sample was exposed to air. Another mechanism of molybdenum oxide formation is the oxidation of the surface of the Mo particles that were uncoated with the MoS*_x_*_~2+δ_ thin shell. The former mechanism seems to be more likely, because amorphous MoS*_x_* films obtained by electrochemical deposition (i.e., those without Mo nanoparticles) also undergo a slow transformation from Mo^4+^ to Mo^6+^ under atmospheric conditions [18]. This process could have partially occurred between the preparation and characterization of these MoS*_x_* thin films.

The XPS spectrum of S 2p for the same MoS*_x_*_~2+δ_/Mo film is shown in Figure 6c. This figure reveals the presence of different S ligands that were considered in the Mo_3_-S cluster-based model of amorphous MoS*_x_*. The S ligands with an S 2p_3/2_ peak at 162.3 eV were assigned to the S_2_^2−^ terminal or unsaturated S^2−^ entities in the amorphous MoS*_x_* and S^2−^ in the crystalline MoS_2_. The S 2p_3/2_ peak at 163.7 eV corresponded to the bridging S_2_^2−^ and apical S^2−^ ligands of the Mo_3_-S cluster [17,18,37,44,45]. This result agrees well with the abovementioned Raman spectra of the MoS*_x_*_~2+δ_/Mo films (Figure 6a). The oxidative process of the MoS*_x_*_~2+δ*/*_Mo film in the air involved, to some extent, the S atoms. Consequently, a broad band at ~169 eV appeared on the XPS spectrum, and this binding energy was assigned to the S-O bonds [17,18].

Quantitative compositional analysis by XPS indicated that the S content in the surface layer of the MoS*_x_*_~2+δ_/Mo films was slightly higher than in the bulk of the films. The *x* value measured by XPS was 5–10% larger than that measured by RBS. This could be due to the adsorption of sulfur atoms on the surface of the films from the residual atmosphere in the deposition chamber after the PLD process finished. A similar result was earlier revealed in Reference [35]. The O concentration in the surface layer of the films did not exceed 10 at % after prolonged exposure in the air. The O concentration was reduced to 3 at % after ion sputtering of the layer of surface contamination for 30 s.

### 3.3. Deposition of the MoS_x~3+δ_ Films by off-Axis PLD

**Figure 7:**
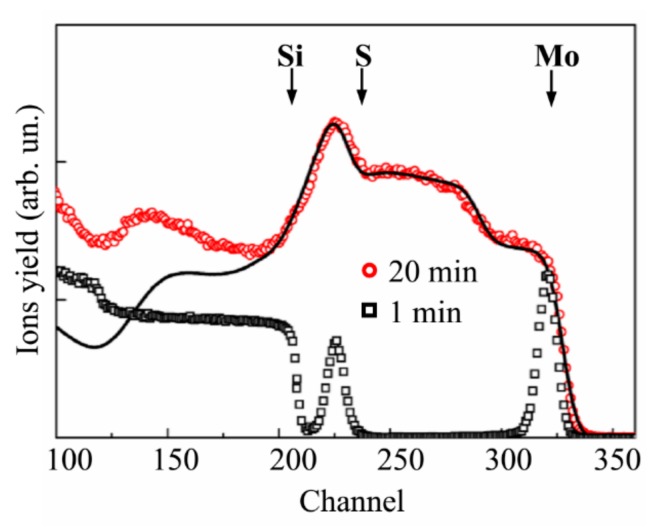
RBS spectra for MoS*_x_*_~3+δ_ films obtained by off-axis PLD on the Si/SiO_2_ substrates in Ar at a pressure of 8 Pa for 1 and 20 min. The solid lines indicate the model/fitted RBS spectrum for the corresponding experimental spectrum.

Figure 7 shows the RBS spectrum of a thin MoS*_x_* film deposited on a Si/SiO_2_ substrate for 1 min using the off-axis mode of PLD in Ar at a pressure of 8 Pa. Fitting of the spectrum revealed that the experimental RBS spectrum coincided well with the model RBS spectrum that was calculated for a continuous/smooth thin film with a composition of MoS*_x_*_~3.9_ (results not shown). The thin film contained ~2.6 × 10^16^ Mo atom/cm^2^ (~4 μg/cm^2^) and ~1 × 10^17^ S atom/cm^2^ (6 μg/cm^2^). Increases in deposition time caused increases in catalyst loading with a sublinear dependence. To fit the experimental RBS spectrum from a thicker film obtained by off-axis PLD for 20 min, a two-layer film model was necessary (Figure 7). A layer of MoS_4.1_ was formed on the surface of this film, and a sublayer was composed of MoS_3.8_. The film contained ~4.2 × 10^17^ Mo atom/cm^2^ (65 μg/cm^2^) and ~1.6 × 10^18^ S atom/cm^2^ (98 μg/cm^2^). Thus, the catalyst loading was increased to 163 μg/cm^2^.

RBS studies have shown that with increasing target ablation time, the composition of the laser plume can be altered to some extent. As a rule, the surface composition and roughness of the transition metal dichalcogenide target are significantly modified by prolonged pulsed laser irradiation [41]. However, an accumulation of sulfur vapor in the film production chamber is also possible with an increase in deposition time. The effects of these factors were also observed during the formation of a thicker MoS*_x_*/Mo film by off-axis PLD.

Notably, upon calculating the model spectrum of a thicker film, the spectrum was found to be well-matched to the experimental spectrum in the channel range that corresponded to He ion scattering from Mo and S atoms. The carbon concentration in the film was set to no more than 20 at %. This value was determined by the X-ray energy dispersive spectroscopy of this film (results not shown). A disagreement between the model and experimental spectra was observed in the range of channels (less than 200), in which the ions yielded from the Si/SiO_2_ substrate were accumulated. This result could be due to the structural features of a thicker film. Below, we demonstrate that the microstructure of the film (micro-crack formation) allowed for the “channeling” of ions through the film. It is difficult to achieve a good fit result for either the film or the substrate under these conditions.

**Figure 8:**
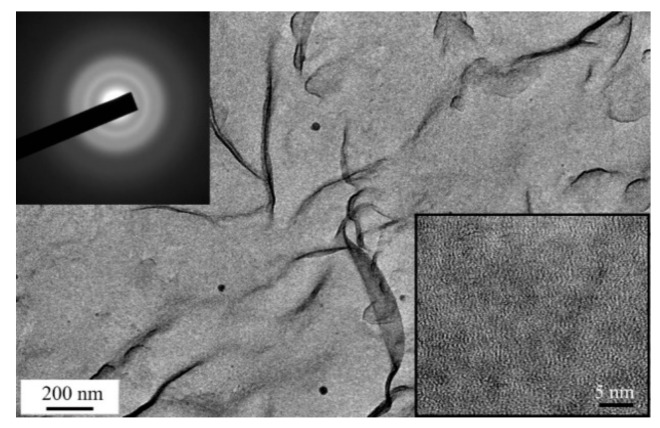
TEM image and SAED pattern of thin MoS*_x_*_~3+δ_ thin film obtained by off-axis PLD in Ar at a pressure of 8 Pa. The bottom insert shows the high resolution TEM image of the film.

The TEM and SAED studies revealed that the use of the off-axis PLD mode substantially decreased the Mo nanoparticle deposition on the surface of the MoS*_x_*_~3+δ_ film (Figure 8). The TEM image contrast and SAED pattern indicated the amorphous and quite homogeneous structure of the thin film. At low magnification, only individual Mo nanoparticles were observed on the TEM image. The high resolution TEM image of the MoS*_x_*_~3+δ_ film differed from that of the MoS*_x_*_~2+δ_/Mo film in terms of a pronounced contrast that contained nanosized threads of dark and light tones. This result could be due to the different natures of the local packings of atoms in the films obtained by on- and off-axis PLD.

**Figure 9:**
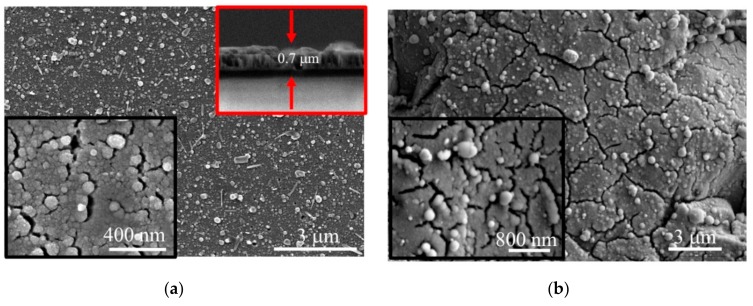
SEM images (two magnifications) of MoS*_x_*_~3+δ_ films obtained on (**a**) Si/SiO_2_ and (**b**) glassy carbon substrates by off-axis PLD in Ar at a pressure of 8 Pa for 20 min. The top inset in (**a**) shows a cross-sectional image of the film.

SEM studies of the MoS*_x_*_~3+δ_ films revealed the formation of a relatively dense thin film material with a quite smooth surface, and the morphology was slightly dependent on both the deposition time and the nature of the substrate (Figure 9). The main effect on the growth of the films during the off-axis PLD was the fragmentation of the films, which caused the formation of micro-blocks that were separated by grooves (micro-cracks). An SEM study of the cross section of the films revealed that the micro-cracks could have been formed, due to the development of a columnar structure in the films. The columnar units originated on the substrate-film interface and grew up to the surface of the film. This growth was characteristic of chemical compound films with a cauliflower structure in which bushes are formed, due to the deposition of the scattered flux of atoms and/or clusters of atoms of the laser plume [46].

The separate rounded particles of submicron size present on the surfaces of these films (Figure 9) could have been formed by the deposition of Mo nanoparticles that subsequently grew in size, due to the deposition of a vapor. The needle-like submicroparticles that appeared on the film surface could have been formed, due to the spreading and solidification of larger liquid droplets that were ejected from the target at high speed and slid over the film surface.

**Figure 10:**
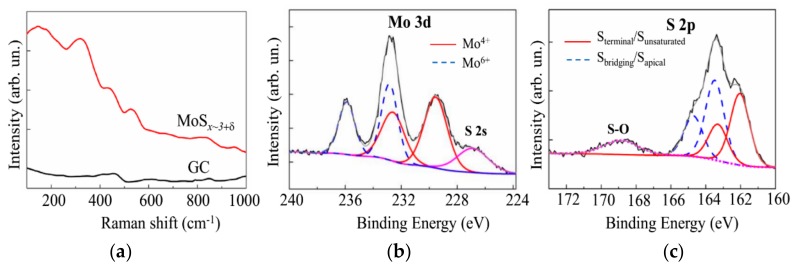
(**a**) Raman spectra for the GC substrate, with and without the MoS*_x_*_~3+δ_ thin film catalyst obtained by off-axis PLD. (**b,c**) XPS spectra of Mo 3d and S 2p, respectively, measured on the surface of the MoS*_x_*_~3+δ_ catalyst after prolonged exposure in the air.

The results of the MRS and XPS studies, shown in Figure 10, did not reveal essential differences in the local structural or chemical states of the catalysts formed by the on- and off-axis PLD. Indeed, the Raman spectra of the MoS*_x_*_~3+δ_ catalysts were wholly like those of the MoS*_x_*_~2+δ_/Mo catalysts (Figure 6a). The XPS Mo 3d spectrum for the catalyst obtained by off-axis PLD for 20 min consisted of two doublets that corresponded to Mo^4+^ and Mo^6+^. Regarding the XPS S 2p spectrum of the MoS*_x_*_~3+δ_ catalyst, the intensity of the doublet with high binding energy was greater than that with low binding energy. Similar results were obtained for the MoS*_x_*_~2+δ_/Mo catalyst. However, in the S 2p spectrum of the MoS*_x_*_~3+δ_ film, the relative intensity of the band corresponding to the S-O bonds was noticeably larger than that in the spectrum of the MoS*_x_*_~2+δ_/Mo film. This finding suggests that, at higher S concentrations in the catalyst, not all S atoms formed perfect chemical bonds with Mo or other S atoms included in the Mo_3_-S clusters. The S atoms possessing unsaturated bonds were subject to oxidation in the air environment.

### 3.4. Electrocatalytic Performances of the MoS_x~2+δ/_Mo and MoS_x~3+δ_ Films Prepared by on-Axis and off-Axis PLD

**Figure 11:**
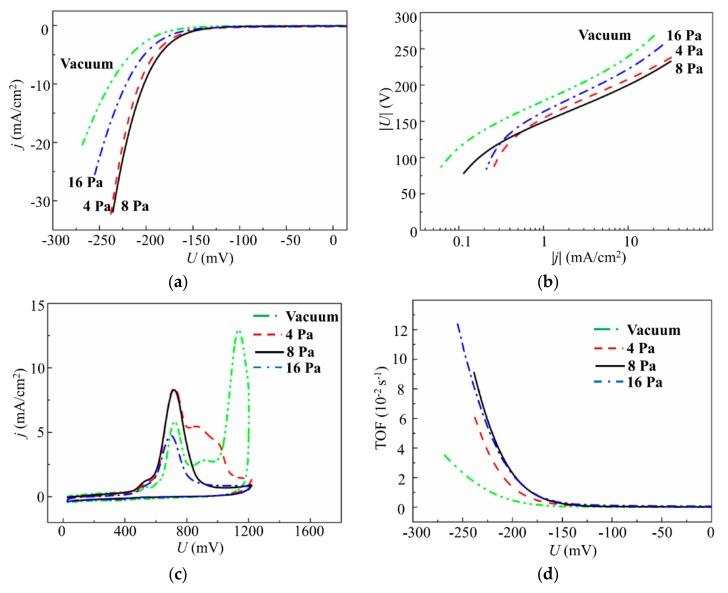
(**a**) Polarization curves. (**b**) Tafel plots. (**c**) Anodic cyclic voltammograms. (**d**) Turnover frequency (TOF) dependence on voltage for the MoS*_x_*_~2+δ_/Mo catalysts obtained on GC substrate by on-axis PLD in a vacuum and at different Ar pressures for 4 min.

Figure 11 shows the results of an electrochemical study of the dependence of the electrocatalytic properties of the MoS*_x_*_~2+δ_/Mo films on the buffer Ar gas pressure. The results of LV measurements indicate that the smallest overpotential of HER was found for films deposited at pressures of 4 and 8 Pa (Figure 11a). A current density of 10 mA/cm^2^ was achieved at a voltage of *U*_10_ ~206 mV. The Tafel slope was ~53.6 eV/dec. The MoS*_x_*_~2+δ_/Mo films deposited in a vacuum and at a higher Ar pressure (16 Pa) required much more overvoltage to achieve a current density of 10 mA/cm^2^, and their Tafel slope was as large as 56.7 mV (Figure 11b). Additional anodic CV measurements and TOF calculations (Figure 11c,d) revealed that the relatively good performance of the catalyst, deposited at a pressure of 4 Pa, was caused by a larger loading compared to that for the catalyst obtained at 8 Pa. Indeed, for the film obtained at 8 Pa, the TOF value at the voltage of −200 mV was ~0.023 s^−1^, and for the film deposited at 4 Pa, the TOF was ~0.014 s^−1^. The films deposited in Ar at 16 Pa also had relatively large TOF (~0.023 s^−1^). However, at the pressure of 16 Pa, the MoS*_x_*_~2+δ_/Mo film deposition rate was the lowest. These results were used to choose the Ar pressure of 8 Pa for the PLD of amorphous MoS*_x_* films in the present work.

Comparison of the shapes of the anodic CV curves (anodic stripping voltammograms) for the MoS*_x_*_~2+δ_/Mo films deposited at different Ar pressures indicated that an increase in Ar pressure of up to 8 Pa led to the formation of a curve in which a broad peak at ~700 mV was dominant (Figure 11c). Other peaks at higher voltages, which were present on the CV curves for the MoS*_x_*_~2+δ_/Mo films deposited in a vacuum and at Ar at 4 Pa, disappeared. This finding suggests that Ar pressure increases during the on-axis PLD, resulting in the formation of a homogeneous local structure of MoS*_x_*_~2+δ_ (0.7 ≤ δ ≤0.9) catalysts, in which all active sites possess an identical nature and participate in the HER. Notably, the oxidation of active sites in the amorphous MoS*_x_* catalysts obtained by electrodeposition was registered at ~900 mV [18,37]. This finding indicates a possible difference in the local atomic structure of the active sites formed during the PLD and electrochemical deposition of amorphous MoS*_x_* films.

**Figure 12:**
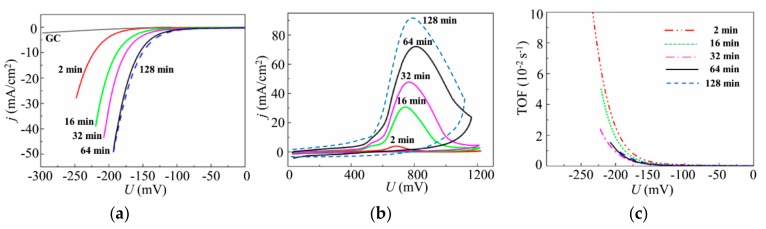
(**a**) Polarization curves. (**b**) Anodic cyclic voltammograms. (**c**) TOF dependence on voltage for the MoS*_x_*_~2+δ_/Mo catalysts obtained on the GC substrate by on-axis PLD in Ar at 8 Pa for different deposition times.

Figure 12 shows the results of the studies of the electrocatalytic properties of the MoS*_x_*_~2+δ_/Mo films that were deposited by on-axis PLD onto the glassy carbon in Ar at 8 Pa for different times. The polarization curves (Figure 12a) show the benchmark activities of the MoS*_x_*_~2+δ_/Mo films, where both the apparent geometric area and the catalyst loading are known. The LV measurements indicated that a noticeable catalytic effect from a thin film was observed for very short deposition times (~2 min). With MoS*_x_*_~2+δ_/Mo catalyst loading of 6.8 μg/cm^2^, an overpotential of −222 mV was required to achieve a current density of 10 mA/cm^2^. With an increase in the deposition time to 64 min, the *U*_10_ value decreased (in absolute value) to −154.5 mV. A significant time increase up to 128 min caused only a slight decrease of the *U*_10_ to −150 mV. The Tafel slopes of the linear portions of the LV curves decreased from 53.7 to 50 mV/dec.

All anodic CV curves, shown in Figure 12b, had approximately identical shapes in which a broad peak dominated. This peak shifted from ~700 to ~800 mV under loading growth. The intensity of the peak revealed an outrunning growth with catalyst loading increase that caused a change of the TOFs for these films. The highest TOF value, −200 mV (equal to 0.026 s^−1^), was found for the catalyst with minimal loading. As the loading increased, the TOFs decreased, and the TOF values were in the range of 0.01 ± 0.002 s^−1^ for the films with higher loadings.

**Figure 13:**
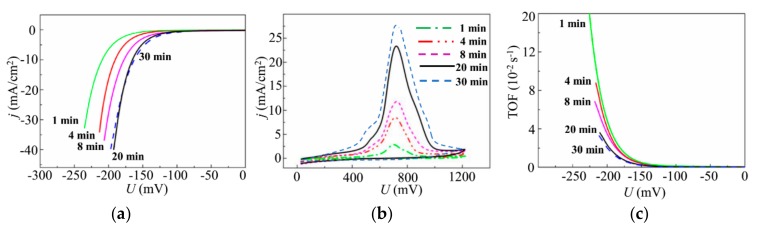
(**a**) Polarization curves. (**b**) Anodic cyclic voltammograms. (**c**) TOF dependence on voltage for the MoS*_x_*_~3+δ_ catalysts obtained on the GC substrate by off-axis PLD in Ar at 8 Pa for different deposition times.

The results of the study of the electrocatalytic properties of the MoS*_x_*_~3+δ_ films deposited on glassy carbon by off-axis PLD in Ar at 8 Pa for different times are shown in Figure 13. For the lowest deposition time of 1 min (MoS*_x_*_~3+δ_ catalyst loading 10 μg/cm^2^), an overpotential of −209 mV was required to achieve a current density of 10 mA/cm^2^. An increase in the deposition time, up to a certain point (20 min), caused a monotonic decrease of the *U*_10_ to −165.5 mV (Figure 13a). The Tafel slope decreased from 48.2 to 44.4 mV/dec. Further time increases caused only a slight decrease of *U*_10_ to −162 mV. The Tafel slopes of 50 and 44.4 mV/dec indicated that the HER was actually proceeded by a mechanism that was identical for the films obtained by on- and off-axis PLD.

The LV measurements indicated that the maximum achievable catalytic performance for the MoS*_x_*_~3+δ_ films was less than that for the MoS*_x_*_~2+δ_/Mo films. This finding contradicted the results of the TOF measurements, which revealed that the TOFs were higher for the MoS*_x_*_~3+δ_ films (Figure 13c) than for the MoS*_x_*_~2+δ_/Mo films (Figure 12c). Indeed, the highest TOF of ~0.05 s^−1^ (at −200 mV) was found for the thinnest MoS*_x_*_~3+δ_ film that was deposited for 1 min. An increase in loading caused a decrease in the TOF of the MoS*_x_*_~3+δ_ film, which was ~0.024 s^−1^ for the film deposited for ≥20 min. The larger TOFs for the MoS*_x_*_~3+δ_ films were due to both a lower current density during anodic striping CV and a narrower shape of the main peak, which is ascribable to the active site oxidation (Figure 13b). The narrower CV peak located at ~710 mV indicated a uniform chemical state of the atoms in the local areas of the catalytically active sites on the surfaces of the MoS*_x_*_~3+δ_ films.

## 4. Discussion

**Figure 14:**
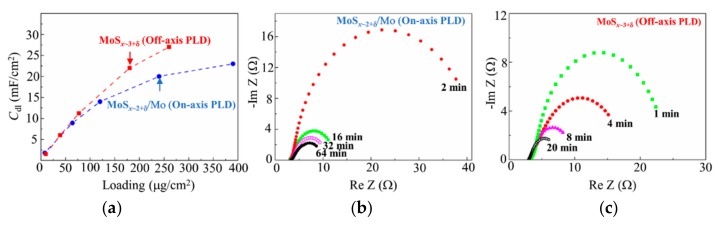
(**a**) Dependences of *C*_dl_ on the loading for the MoS_x_-based catalysts obtained by on- and off-axis PLD. (**b**, **c**) Nyquist plots of the electrochemical impedance spectroscopy (EIS) measured for these catalysts obtained with different deposition times. The arrows indicate in (**a**) the optimal loadings of the catalysts.

For both PLD deposition modes, an increase in catalyst loading caused a sublinear increase in the double-layer capacities of the obtained catalysts. However, at higher loadings, the *C*_dl_ for the MoS*_x_*_~3+δ_ films exceeded that of the MoS*_x_*_~2+δ_/Mo films (Figure 14a). Thus, for the optimal loadings of 240 µg/cm^2^ (MoS*_x_*_~2+δ_/Mo catalyst) and 163 µg/cm^2^ (MoS*_x_*_~3+δ_ catalyst), the *C*_dl_ values were 20 and 22 mF/cm^2^, respectively. These findings could be because there were significant contributions of heavy Mo particles to the loadings of the MoS*_x_*_~2+δ_/Mo films. For both films, the contents of S atoms that could form catalytically active sites (as S_2_^2−^ units) in these types of catalysts were approximately equal to 1.5 ± 0.1 × 10^18^ cm^−2^.

Double layer capacitances are directly proportional to the effective electrochemical surface areas of the catalysts. However, the proportionality coefficient may depend on the nature of the catalyst-electrolyte interface. It is reasonable to assume that the specific capacities of two structures, i.e., semiconductor (MoS*_x_*_~3+δ_)-electrolyte and metal (Mo)-semiconductor (MoS*_x_*_~2+δ_)-electrolyte, might differ. Thus, it is difficult to correctly compare the effective electrochemical surface areas of the catalysts obtained by on- and off-axis PLD using the results of the *C*_dl_ measurements. Notably, the *C*_dl_ increased, even with an increase in loading past the optimal point. This allowed us to suggest that, under high loading, the entire electrochemical active surface area is not effectively involved in the HER. This supposition could be due to the fact that other mechanisms also influence the hydrogen evolution. Both the efficient transfer/diffusion of reagents to active sites and conditions for the free removal of molecular hydrogen are required [47,48,49]. The porous structures of the MoS*_x_*_~2+δ_/Mo films, with greater physical surface area, are expected to provide such conditions. Analysis of the anodic stripping voltammograms revealed that, on the physical surfaces of the MoS*_x_*_~2+δ_/Mo films, there were many more active centers than on the surfaces of the MoS*_x_*_~3+δ_ films (Figure 12b).

EIS revealed that the lower TOFs of the MoS*_x_*_~2+δ_/Mo films could have been caused not only by lower S concentrations in the catalytic MoS*_x_*_~2+δ_ films, but also by increased resistivities to the electric current transport through the hybrid layers. Indeed, the increase in load caused a monotonic decrease in the electrical resistances of the MoS*_x_*_~2+δ_/Mo and MoS*_x_*_~3+δ_ films (Figure 14b,c). However, with optimal loading, the resistances to current transport of the MoS*_x_*_~2+δ_/Mo and MoS*_x_*_~3+δ_ films were ~11 and ~7 Ω, respectively. As is known, the resistivity of a catalytic film has an important influence on the kinetics of the HER [50]. The increased resistivities of the MoS*_x_*_~2+δ_/Mo films could be caused by the hybrid structures of these films. Although metallic Mo is a good current conductor, in a hybrid structure, the electric current mainly passes through Mo-MoS*_x_*_~2+δ_ interfaces, which could produce a barrier effect (i.e., a Schottky barrier) for electron transport [51].

Long-term stability is an important aspect when evaluating the performance of an electrocatalyst. For the on- and off-axis PLD catalysts, nearly identical LV curves were detected after the first and 2000th cycles of CV testing. These results indicate no loss of catalytic performance and the remarkable stabilities of the electrocatalysts prepared on the GC substrate by the two PLD modes. Notably, the HER performances of both types of catalysts obtained with the two modes of PLD were quite high compared to other state-of-the-art nonprecious catalysts in acidic solutions. Comprehensive and updated information about the HER performances of amorphous MoS*_x_*-based catalysts can be found in the literature [17,18,37].

To assess the potential feasibility of practical application of on-axis and off-axis PLD methods, the distribution of the thickness and composition of the MoS*_x_* films on the substrate should be considered. In the case of the vacuum on-axis PLD of the MoS*_x_*_~2+δ_/Mo film on the substrate installed at 3.5–5 cm from the MoS_2_ target, almost all the laser ablated material is deposited on a substrate with a diameter of ~4–6 cm. The bell-shaped distribution of the film thickness is formed over the substrate surface [24]. The use of a buffer gas can improve the uniformity of film deposition, due to scattering of the laser plume (see, for example, Reference [52]), but the amount of the deposited material will be decreased. Analysis of Figure 3b revealed that at an optimal (for obtaining the highest HER efficiency of MoS*_x_*_~2+δ_/Mo film) Ar gas pressure, the amount of material deposited by on-axis PLD was decreased by ~6 times in comparison with the deposition by vacuum on-axis PLD.

The studies have shown that the atomic flux ablated by the laser from the MoS_2_ target and scattered by Ar gas will not be wasted. The deposition of the scattered atomic flux also makes it possible to obtain rather effective MoS*_x_*_~3+δ_ electrocatalysts by off-axis PLD. For example, two substrates can be installed in parallel on opposite sides of the axis of expansion of the laser plume. Thus, most of the ablated material will be used to obtain the MoS*_x_*_~3+δ_ electrocatalyst. Studies of the film composition/thickness on the substrate at various distances from the target and the development of algorithms for moving/rotating substrates during the off-axis PLD processes will result in the preparation of rather uniform distributions of the catalytic MoS*_x_*_~3+δ_ films over the substrates with surface areas of ~10 cm^2^.

A technical solution involving a combination of two methods of PLD may be promising. The on-axis PLD will cause the formation of a porous structure of the catalytic MoS*_x_*_~2+δ_/Mo layer, and off-axis PLD will result in the formation of a highly-effective MoS*_x_*_~3+δ_ thin-film electrocatalyst on the surface of the porous MoS*_x_*_~2+δ_/Mo layer.

## 5. Conclusions

The use of two modes of PLD from a MoS_2_ target allowed us to obtain amorphous MoS*_x_*-based catalysts with different compositions and morphologies. To obtain a high-quality MoS*_x_*-based catalyst, it is important to choose the optimal buffer gas pressure during the PLD. During the deposition by traditional on-axis PLD, MoS*_x_*_~2+δ_/Mo films with hybrid porous structures and rather high concentrations of sulfur (δ ~0.7) in the catalytically active MoS*_x_*_~2+δ_ nanophase were obtained. Submicron and nanosized Mo-based particles were formed during the pulsed laser ablation of the target, and they were then incorporated into the bulk of the catalytic coatings deposited on the GC substrate. The hybrid structure somewhat slowed down the electric current transport through the deposited layer; however, this provided a greater physical surface area, and consequently, a greater number of active sites for HER. The porosity/morphology of the hybrid catalyst facilitated the effective growth of the exposed surface area with the increase in the MoS*_x_*_~2+δ_/Mo catalyst loading. This led to the possibility of achieving a higher HER performance at the optimal catalyst loading (~240 μg/cm^2^) via the use of on-axis PLD compared to what could be achieved with off-axis PLD. To realize a current density of 10 mA/cm^2^ on the GC substrate, the lowest overvoltages were −150 and −162 mV for the films obtained by on- and off-axis PLD, respectively.

The mode of off-axis PLD differs from the mode of on-axis PLD in terms of a higher deposition rate for catalytic MoS*_x_*_~3+δ_ films and a larger S concentration in the amorphous MoS*_x_*_~3+δ_ (δ ~0.8–1.1) phase. The depositions of Mo and S atomic fluxes scattered on Ar molecules facilitated the growth of the relatively dense structures of these films, which contributed to improved electric current transport through the deposited layer. This fact, combined with the greater S concentration, resulted in higher TOFs for the MoS*_x_*_~3+δ_ than the MoS*_x_*_~2+δ_/Mo films. However, the growth of the loading of these relatively dense catalytic films that possessed smoother surfaces did not effectively increase the numbers of active sites exposed in electrolyte for HER. The HER performance of the MoS*_x_*_~3+δ_ films obtained with off-axis PLD was saturated at a loading of ~163 μg/cm^2^.
